# Heterogeneous pattern of gene expression driven by *TTN* mutation is involved in the construction of a prognosis model of lung squamous cell carcinoma

**DOI:** 10.3389/fonc.2023.916568

**Published:** 2023-03-23

**Authors:** Zhao Liu, Xiaowen Zhao, Ruihong Wang, Xiangyue Tang, Yuxiang Zhao, Guanghui Zhong, Xin Peng, Chunlin Zhang

**Affiliations:** ^1^ Ningbo Municipal Hospital of Traditional Chinese Medicine (TCM), Affiliated Hospital of Zhejiang Chinese Medical University, Ningbo, China; ^2^ United New Drug Research and Development Center, Biotrans Technology Co., LTD., Ningbo, China; ^3^ Institute of Bioengineering, Biotrans Technology Co., LTD., Shanghai, China; ^4^ Qingdao Central Hospital, The Second Affiliated Hospital of Medical College of Qingdao University, Qingdao, China

**Keywords:** bioinformatics, lung squamous carcinoma, TTN, gene mutation, prognostic survival

## Abstract

**Objective:**

To investigate the impact that *TTN* mutation had on the gene heterogeneity expression and prognosis in patients with lung adenocarcinoma.

**Methods:**

In this study, the Cancer Genome Atlas (TCGA) dataset was used to analyze the *TTN* mutations in lung adenocarcinoma. Lung adenocarcinoma data was collected from the TCGA database, clinical information of patients was analyzed, and bioinformatics statistical methods were applied for mutation analysis and prognosis survival analysis. The results were verified using the GEO dataset.

**Results:**

The incidence of *TTN* mutations in lung adenocarcinoma was found to be 73%, and it was related to the prognosis of lung adenocarcinoma. Ten genes were screened with significant contributions to prognosis. A prognosis model was constructed and verified by LASSO COX analysis in the TCGA and GEO datasets based on these ten beneficial factors. The independent prognostic factor H2BC9 for *TTN* mutation-driven gene heterogeneity expression was screened through multi-factor COX regression analysis.

**Conclusion:**

Our data showed that the gene heterogeneity expression, which was driven by *TTN* mutations, prolonged the survival of lung adenocarcinoma patients and provided valuable clues for the prognosis of *TTN* gene mutations in lung adenocarcinoma.

## Introduction

Lung cancer remains the leading type of cancer-related death, and gene mutations have a very important impact on prognosis in NSCLC (1). TTN and TP53 are two of the most commonly mutated genes in NSCLC samples from The Cancer Genome Atlas (TCGA) official website. TP53 plays a crucial biological role in the development of various tumors and has received widespread attention (2–4). Co-mutation analyses of TP53 and cancer driver genes in clinical studies can often be used to provide specific molecular typing for molecularly targeted therapies (5). The coding gene TTN, also known as cardiomyopathy dilated 1G, is the longest-reported gene to date (6). TTN has a high mutation rate in several types of tumor tissue (7). However, TTN is controversial as it is considered a tumor-associated gene. This controversy focuses on the large and complex DNA structure and the false-positive results associated with tumor heterogeneity. In addition, the biological function of TTN mutations in the development of cancer has been questioned, as TTN mutations have been strongly associated with cardiac and skeletal muscle diseases but rarely with oncological diseases (6, 8, 9). To date, only a few studies have specifically explored the relevance of TTN to tumors. The complexity of the TTN gene structure compared to TP53 and the aforementioned controversies regarding TTN have greatly hindered researchers from analyzing this gene in depth. To date, studies related to TTN mutations in tumors are rare.

Using the TCGA database, this paper focuses on the correlation between TTN mutations and the clinicopathological characteristics of patients with LUSC and the impact of these mutations on the survival of these patients. It also focuses on the impact of TTN co-mutations on the prognosis survival of these patients to identify the main prognostic factors that affect TTN mutations in LUSC and thus provide new possible treatment targets for the disease. The main prognostic factors affecting TTN mutations in LUSC are SLC34A2, BARX1, and HOXC10. In this study, we elucidated the prognostic impact of heterogeneous gene expression caused by TTN mutations on LUSC and screened the main prognostic factors of heterogeneous gene expression driven by TTN mutations using the least absolute shrinkage and selection operator (LASSO) Cox analysis. The results could provide new possible targets for the prevention and treatment of LUSC.

## Methods and materials

### Data sources

RNA sequencing data (level 3), expression profile data, and corresponding clinical information were obtained from a TCGA dataset (https://portal.gdc.com) for 488 patients with LUSC. The GSE73403 datasets was from the Gene Expression Omnibus (GEO) database (https://www.ncbi.nlm.nih.gov/geo).

### Gene mutation analysis

The data of 488 patients with LUSC got from TCGA were analyzed, and somatic mutations in patients with LUSC were visualized using the maftools package in R software.

### Clinical correlation analysis

A univariate analysis of the clinicopathological characteristics was performed using the chi-square test and nonparametric tests. This was followed by screening for statistically significant independent influences associated with *TTN* mutations using binary logistic regression models. P values < 0.05 were considered significant.

### Survival analysis

RNAseq and corresponding clinical information of 393 *TTN* mutations in LUSC were came from TCGA dataset (https://portal.gdc.com). Progression-free survival (PFS), disease-free survival (DFS), and disease-specific survival (DSS) were assessed by overall survival (OS). The impact of *TTN* mutation on the prognostic survival of LUSC was assessed. Survival analyzes were performed using Cox multiple survival regression model analysis as the primary method, and the Kaplan–Meier (KM) method as an adjunct method. The endpoint of OS was defined as the time from randomization to death from any cause. The endpoint of DFS was defined as the time from the start of the initial treatment to the onset of a new tumor-related event or death from any cause. All the above analysis methods were implemented with the R package. P values < 0.05 were considered significant.

### Differential expression analysis

The DESeq2 package of R software was used for expression profiling, differential analysis, gene volcano mapping, and heat mapping. For gene microarray screening, corrected p-values and |log1.5FC| p < 0.05 and |log1.5FC| ≥ 1.0 were calculated for differentially expressed genes. Gene ontology (GO) functional enrichment and Kyoto Encyclopedia of Genes and Genomes (KEGG) pathway analysis enrichment analysis were visualized using the ClusterProfiler package in R software, and a false discovery rate of < 0.01 was considered significant.

### Construction of prognostic signature models

The relationship between prognostic immune-related gene expression and OS was analyzed. A prognostic risk prediction model for LUSC was calculated. Patients with LUSC were divided into high-risk and low-risk groups using the median risk score as the cut-off. KM curves were plotted to compare these groups. Receiver operating characteristic (ROC) survival analysis was performed using the R package SURVIVAL, and decision curve analysis was performed using the rmda package. The association between risk score models and tumor immune-infiltrating cells was also investigated using Spearman correlation analysis, and statistical significance was set to P < 0.05.

## Results

### Mutation landscape in LUSC tissues

**Figure 1:**
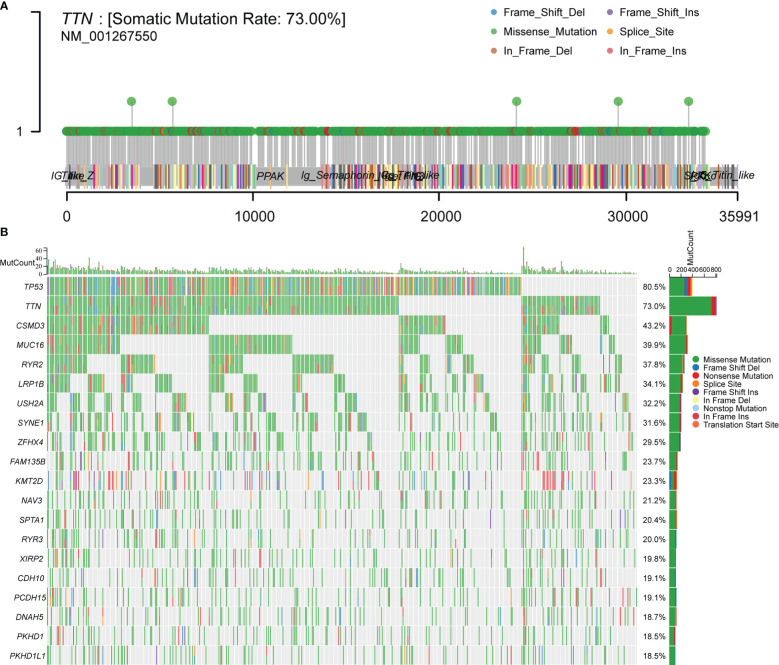
Downloading and visualizing somatic mutations in patients with LUSC using the maftools package in R software for TCGA data. **(A)** Lollipop plot of *TTN* gene mutation distribution with 73% mutation rate in somatic cells. **(B)** Somatic cell landscape of LUSC patient cohort with genes sorted by mutation frequency and samples sorted by disease histology; sidebar plot showing log_10_ transformed *Q*-values estimated by MutSigCV. The waterfall plot shows mutation information for each gene in each sample.

To make sure the frequency of mutations in genes in LUSC, we imported data from the TCGA database for analysis. A total of 488 patients with LUSC were imported, containing 478 samples with detected mutations, of which the mapping samples contained a total of 478 (97.75%). The results showed that the *TTN* mutation rate was 73.00% (**Figures 1A, B**).

### Mutations in the *TTN* gene lead to differences in clinical characteristics of LUSC patients

Subsequently, our univariate analysis of *TTN* gene mutations and clinicopathological characteristics showed that survival status, pN stage, smoking history, and history of neoadjuvant treatment were associated with *TTN* mutations, while gender, age, pathological stage, new tumor event type, and radiation/chemotherapy were not associated with *TTN* mutations in LUSC (**Table 1**).

**Table 1:** Correlation analysis between *TTN* mutation and clinicopathological characteristics of LUSC.

	Character	*TTN* mutant	*TTN* WT	*P*-value
Survival status	Alive	233	45	
	Dead	160	51	0.037
Age	Mean (SD)	66.9 (8.5)	68.1 (9.2)	
	Median [MIN, MAX]	68 [39, 84]	69 [41, 90]	0.243
Gender	Female	96	31	
		297	65	0.148
pT stage	T1	40	8	
	T1a	19	5	
	T1b	35	5	
	T2	128	39	
	T2a	72	14	
	T2b	24	10	
	T3	58	10	
	T4	17	5	0.44
pN stage	N0	248	62	
	N1	106	22	
	N2	32	8	
	N3	5		
	NX	2	4	0.031
pM stage	M0	323	78	
	M1	5		
	M1a	1		
	M1b	1		0.709
pTNM stage	I	2	1	
	IA	72	16	
	IB	113	35	
	II	3		
	IIA	54	10	
	IIB	71	19	
	III	2	1	
	IIIA	54	8	
	IIIB	13	4	
	IV	7		0.657
New tumor event type	Metastasis	25	10	
	Metastasis: Primary	2		
	Metastasis: Recurrence	1	1	
	Primary	7	3	
	Recurrence		5	0.619
Smoking	Non-smoking	10	8	
	Smoking	74	85	0.016
Radiation therapy	Non-radiation	111	25	
	Radiation	14	1	0.435
History of neoadjuvant treatment	Neoadjuvant	1	3	
	No neoadjuvant	391	93	
	Yes, Pharmaceutical Treatment Prior to Resection	1		0.03
Therapy type	Ancillary: Chemotherapy	1		
	Chemotherapy	109	21	
	Chemotherapy:	2	1	
	Chemotherapy: Other. specify in notes:Vaccine	1		
	Chemotherapy: Targeted Molecular therapy	1		
	Chemotherapy: Vaccine	1		
	Other. specify in notes	1		
	Vaccine	1		0.995

### *TTN* mutations are associated with longer survival in LUSC

**Figure 2:**
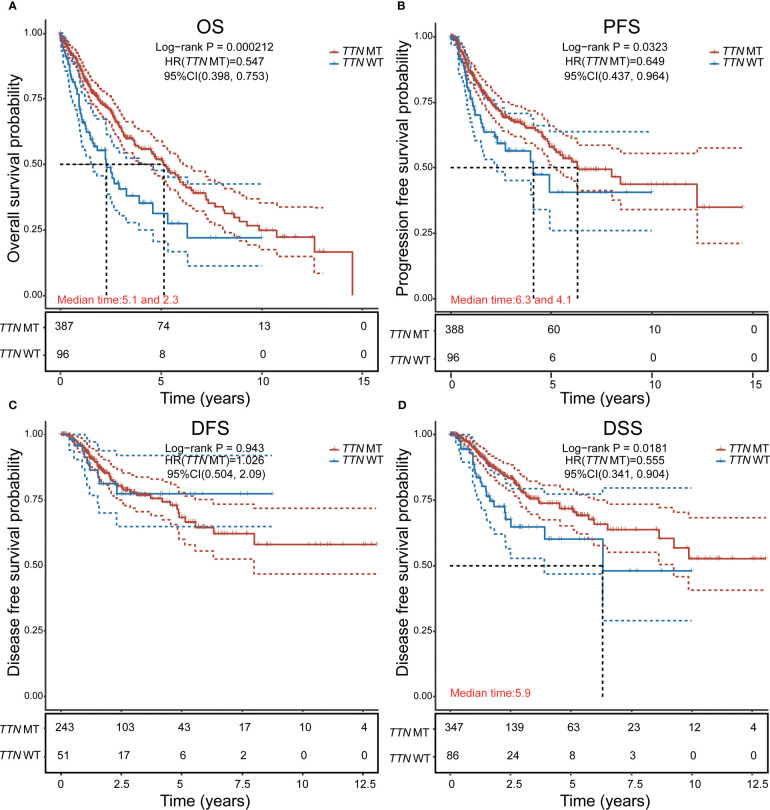
Analysis of the effect of *TTN* mutation on prognostic survival in LUSC using the Cox regression model. **(A)** Overall survival (OS). **(B)** Progression-free survival (PFS). **(C)** Disease-free survival (DFS). **(D)** disease-specific survival (DSS). HR represents the risk coefficient of the high-expression group relative to the samples in the low-expression group; 95% CI represents the HR confidence interval; Median time represents the survival rate of the two groups (high-expression group and low-expression group) at 50% (i.e., median survival time) corresponding to the time in years; and the test between the two groups was performed by log-rank and was statistically significant at P < 0.05.

There were 211 deaths among 489 patients with LUSC (**Table 1**); according to the Cox proportional risk regression model, *TTN* mutation was associated with longer OS (multivariate Cox model P=0.000212, HR=0.547 [95% CI, 0.398–0.753], **Figure 2A**), PFS (multivariate Cox model P=0.0323, HR=0.649 [95% CI, 0.437–0.964], **Figure 2B**), and DSS (multivariate Cox model P=0.0181, HR=0.555 [95% CI, 0.341–0.904], **Figure 2D**), *TTN* mutations was not associated with DFS (**Figure 2C**). Therefore, it can be concluded that *TTN* mutations are associated with survival in LUSC.

### *TTN* mutations in LUSC drive heterogeneous gene expression

**Figure 3:**
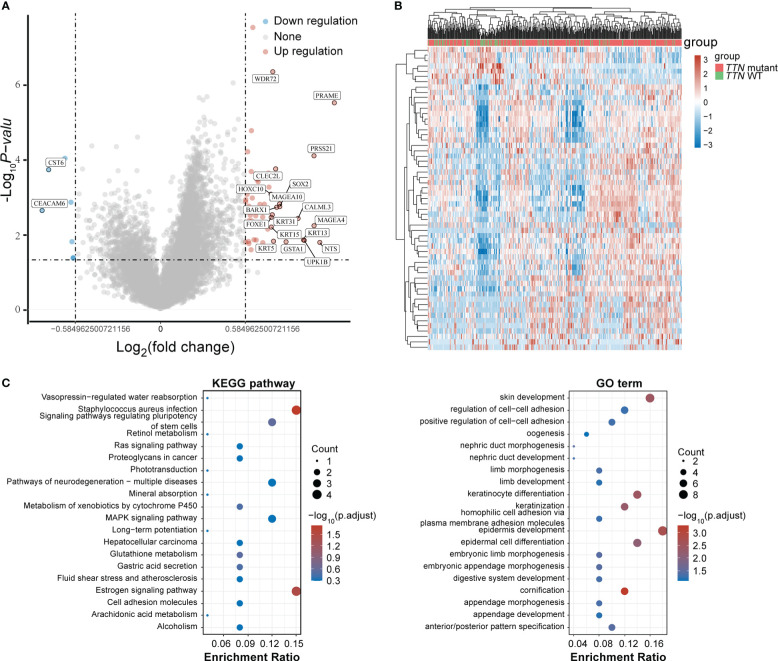
DEGs screening. **(A)** Volcano plot. Red dots represent significantly differentially upregulated genes, blue dots represent significantly differentially downregulated genes, and gray dots represent insignificant genes. **(B)** Heat map of the top 50 significantly DEGs. Different colors represent expression trends in different tissues. **(C)** GO functional enrichment and KEGG pathway analysis of upregulated genes.

To determine the role of gene expression heterogeneity driven by *TTN* mutations in LUSC in the construction of prognostic models, we screened differentially expressed genes (DEGs) in 393 LUSC *TTN* mutant tissues and 96 LUSC tissues in TCGA. A total of 50 up-regulated genes and 7 down-regulated genes were screened, and the volcano are shown in **Figures 3A**, **B** showed the gene expression heat map of each sample, respectively. For DEGs, GO function enrichment and KEGG differential analyzes were performed on DEGs (**Figure 3C**).

### *TTN* mutation prognosis model was built based on 10 prognostically favorable factors

**Figure 4:**
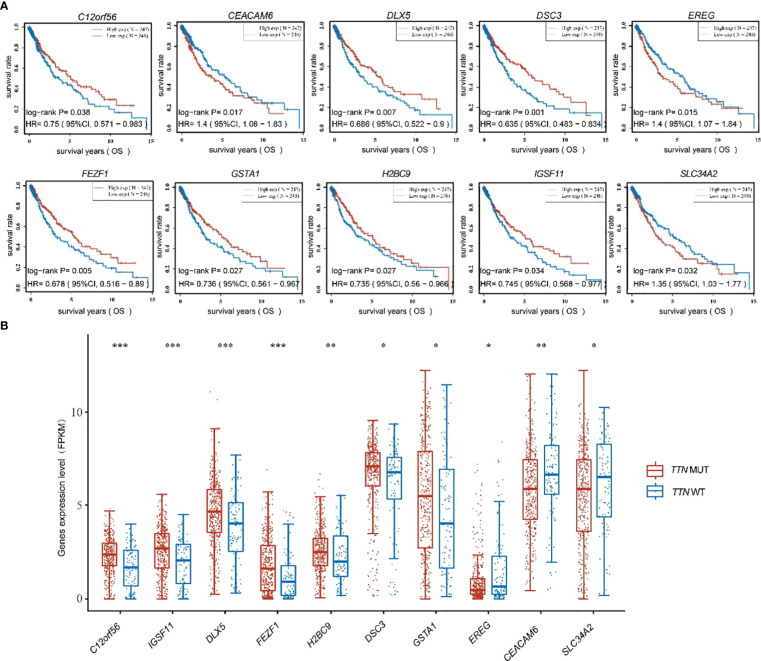
**Figure 4** was shown to present the expression of ten beneficial factors and their impact on patient prognosis. **(A)** The high or low expression of the ten beneficial factors (*C12orf56, DLX5, DSC3, FEZF1, GSTA1, H2BC9, IGSF11, EREG, CEACAM6, SLC34A2*) was demonstrated to have an impact on the prognosis of lung squamous cell carcinoma patients. **(B)** The expression of the ten beneficial factors (*C12orf56, DLX5, DSC3, FEZF1, GSTA1, H2BC9, IGSF11, EREG, CEACAM6, SLC34A2*) in patients with *TTN* mutations and wild-type *TTN* patients was shown to be presented. t test was used for comparison between groups, with * indicating p<0.05, ** indicating p<0.01, and *** indicating p<0.001.

To further discuss the main genes that prolong the survival of lung adenocarcinoma patients with *TTN* mutations, 57 significant differential genes were analyzed for their correlation with the prognosis of lung adenocarcinoma patients, and 10 significant differential genes with prognostic differences were obtained. Among them, low expression of *C12orf56, DLX5, DSC3, FEZF1, GSTA1, H2BC9, IGSF11* and high expression of *EREG, CEACAM6, SLC34A2* in lung adenocarcinoma were found to predict poor prognosis for patients (**Figure 4A**). The results of differential expression analysis showed that *C12orf56, DLX5, DSC3, FEZF1, GSTA1, H2BC9, IGSF11* were highly expressed in *TTN* mutation patients, and *EREG, CEACAM6, SLC34A2* were lowly expressed in *TTN* mutation patients (**Figure 4B**). These data indicate that these 10 significant differential expression prognostic genes are beneficial factors for the prognosis and survival of patients with prolonged *TTN* mutations.

**Figure 5:**
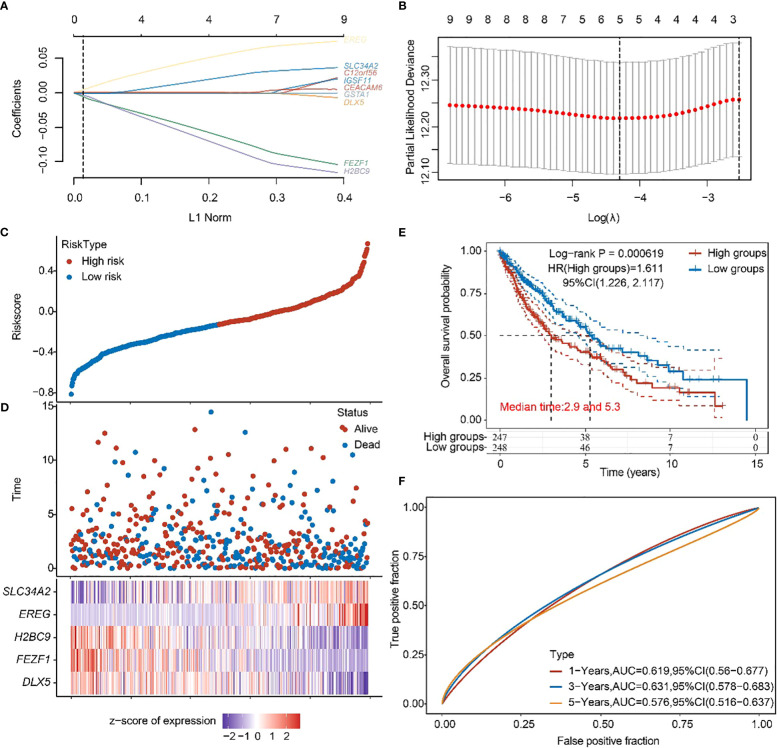
Construction of prognostic features of *TTN* mutation DEGs in LUSC. **(A, B)** Distribution of LASSO coefficients of DEGs to obtain the adjustment parameter. λ.min = 0.0329, the vertical black dashed line in **B** defines the optimal λ value. **(C)** Distribution of risk score of *TTN* mutation in LUSC. **(D)** Survival status and duration of *TTN* mutation in LUSC patients. **(E)** KM survival curves for high- and low-risk groups. **(F)** 1-year, 3-year, and 5-year ROC curves.

Subsequently, a prognostic feature was established based on the 10 beneficial factors using LASSO Cox analysis (**Figures 5A, B**). The lung adenocarcinoma patients were divided into high-risk and low-risk groups using the risk score Riskscore = (-4e-04)*DLX5 + (-0.0772)*FEZF1 + (-0.0933)*H2BC9 + (0.0637)*EREG + (0.0298)*SLC34A2 (**Figure 5C**), and their survival status was displayed in **Figure 5D**. The difference in survival between the high-risk and low-risk groups was shown in **Figure 5E** (P=0.00619). Prognostic survival prediction was performed for 1 year, 3 years, and 5 years, and the prognostic model showed good sensitivity and specificity (**Figure 5F**).

### Validation of the constructed prognostic model by an external independent dataset

**Figure 6:**
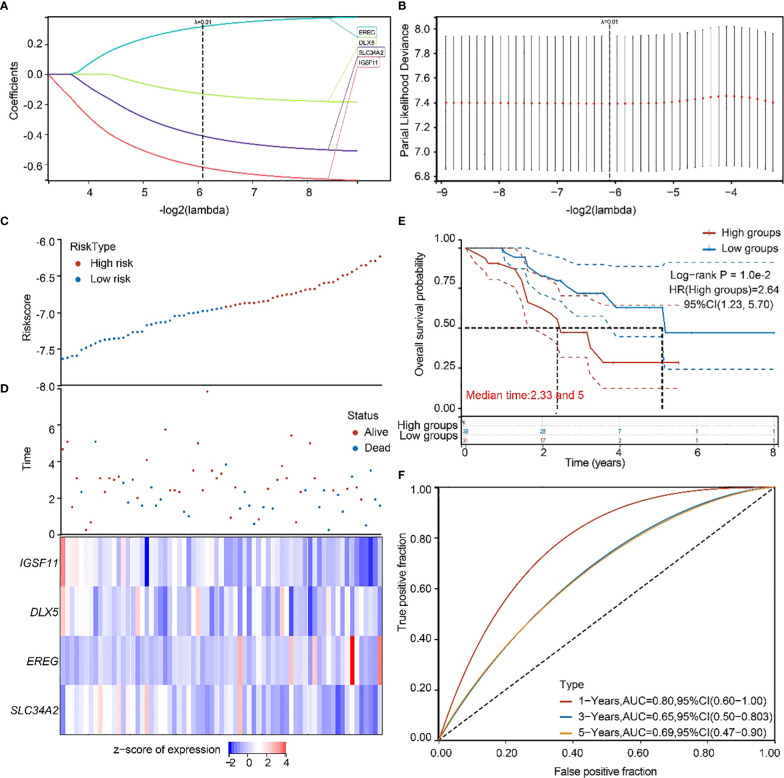
GEO independent dataset to validate the risk model. **(A, B)** Distribution of LASSO coefficients in the GSE73403 datasets, obtained by adjusting the parameters λ.min = 0.0146. **(C, D)** Distribution of risk scores of the constructed models for the GSE73403 datasets. **(E)** KM survival curves for the high-and low-risk groups. **(F)** 1-year, 3-year, and 5-year ROC curves.

To validate the accuracy of our screened independent prognostic factors, we constructed risk prognosis models in GSE73403 datasets and obtained. RiskScore=(-0.61828)*IGSF11+(-0.13167)*DLX5+(0.31607)*EREG+(-0.41127)*SLC34A2 risk assessment models (**Figures 6A, B**), which divided patients into two groups of high risk and low risk (**Figures 6C, D**), with significant differences in prognosis (**Figure 6E**). Prognostic survival was predicted for 1, 3, and 5 years, and this prognostic model showed preferably sensitivity and specificity (**Figure 6F**).

### H2BC9 was identified as an independent prognostic factor for lung squamous cell carcinoma with *TTN* mutation

**Figure 7:**
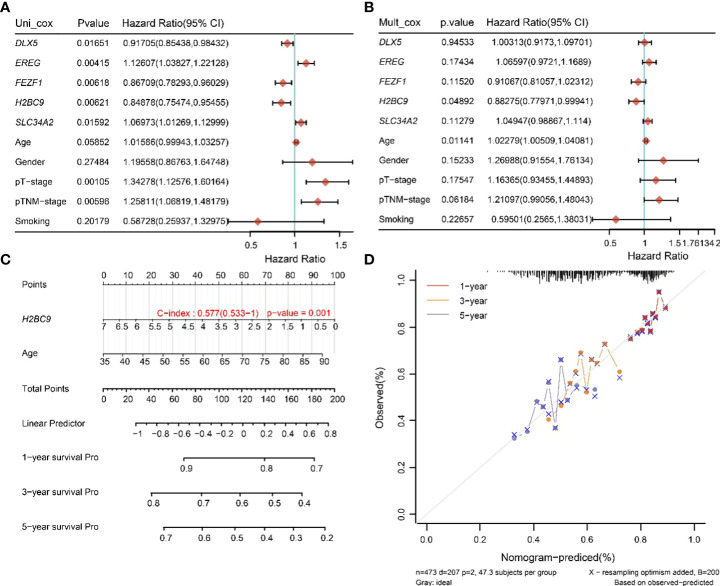
Univariate and multivariate Cox screening prognostic factors. **(A, B)** Analysis of clinical factors and risk scores using univariate Cox analysis, and multivariate Cox analysis was used to analyze significant factors. **(C)** Construction of clinical diagnosis column line graphs based on clinical characteristics and risk scores. **(D)** Calibration plots for predicting recurrence at 1, 3, and 5 years. The x-axis indicates the recurrence probability predicted from the column line graphs, and the y-axis indicates the actual recurrence probability.

To determine the prognostic factors of *TTN* mutations on OS in LUSC, we performed univariate and multivariate Cox regression analysis of prognostic correlates, and the results are shown in **Figures 7A, B**. In the univariate Cox regression analysis, *DLX5, FEZF1, H2BC9, EREG, SLC34A2*, pT-stage and pTNM-stage were significantly correlated with the OS of the *TTN* mutations in LUSC patients (**Figure 6A**). Then, *DLX5, FEZF1, H2BC9, EREG, SLC34A2*, pT-stage and pTNM-stage were analyzed to determine the independent prognostic factors based on a regression analysis of *TTN* mutations in patients with LUSC by multivariateial Cox analysis. H2BC9 was found to be significantly associated with the OS of the *TTN* mutation in the patients (**Figure 7B**). All independent factors were combined to create nomogram prediction plots predicting 1-, 3-, and 5-year OS (**Figures 7C, D**). 

## Discussion

Lung carcinogenesis and progression are complex biological processes with multi-gene involvement and multiple steps. Few driver mutations have been identified in LUSC that can be used as drug targets, and, unlike with lung adenocarcinoma, the process of development does not depend on classical driver mutations, but more on epigenetic alterations (10–12). Therefore, because of its unique molecular pathology, LUSC cannot benefit significantly from molecularly targeted therapies as lung adenocarcinoma can. The TCGA database maps a large number of human tumor genomic variants through genomic analysis techniques. By mining this database with large samples of data, valuable research subjects can be derived and further targeted studies can be conducted. This database can provide a theoretical basis for clinical treatment. In LUSC, exploring the molecular-level determinants of the immunotherapeutic response is challenging, and many studies are underway to address this issue. The results of the TCGA study of mutations in the genome of 178 LUSC patients revealed potential therapeutic targets mainly associated with PI3K/AKT signaling pathways and signaling pathways mediated by receptor tyrosine kinases (RTKs), including *PIK3CA* mutations (16% of cases); *FGFR* amplification or mutations (18% of cases); *PTEN* mutations or deletions (15% of cases); *EGFR* amplification and *PDGFRA* amplification or mutation (9% each); and *ERBB2* amplification, *DDR2* mutation, and *BRAF* mutation (4% each) (13).

In recent years, it has been shown that single or double mutations in *TP53* and *TTN*, two genes with high mutation frequency in NSCLC, have different prognostic effects, and *TTN* gene mutations were significantly associated with LUSC (14). Meanwhile, a study analyzed somatic mutations in LUSC samples based on datasets obtained from TCGA and the International Cancer Genome Consortium (ICGC) database and found that *TTN* mutations were associated with tumor mutational burden (TMB) and positively correlated with LUSC prognosis (15). And in a study by Xie et al. (16), *TTN* with mutations in higher TMB was found to be associated with prognostic outcome, and *TTN* mutations were found to enhance anti-tumor immune responses according to the CIBERSORT algorithm. In the above study, the prognostic impact of *TTN* mutation or double mutation with *TP53* on LUSC was systematically described, and the relationship between *TTN* mutated genes and TMB was analyzed based on TCGA and ICGC databases. In our study, we investigated the prognostic impact of heterogeneous expression of genes due to *TTN* mutations on LUSC from the TCGA database and screened for their independent prognostic factors.

In our study, it was found that the prolonged survival of lung squamous cell carcinoma patients was caused by *TTN* mutation. Based on this, the heterogeneous expression of genes driven by *TTN* mutation was analyzed, and 57 differential genes were identified. After performing a prognostic analysis of the differential genes combined with their expression in *TTN* wild-type and *TTN* mutation patients, 10 genes were found to have contributed significantly to the prognostic potential of prolonged survival. A prognostic model was then constructed based on these 10 prognostic beneficial factors, and a prognostic model with *DLX5, FEZF1, H2BC9, EREG, and SLC34A2* as the main factors was obtained.

*SLC34A2* can be highly expressed in the lung (17, 18). In human lungs, *SLC34A2* is expressed only in type II alveolar epithelial cells (AT-II) and is required for the synthesis of AT-II lung surfactant (19). AT-II cells may transform into cancer stem cells in response to exogenous or endogenous factors and eventually induce oncogenic effects and NSCLC carcinogenesis (20–22). *SLC34A2* may have a physiological function in AT-II, and its mutation or abnormal expression will certainly affect the normal function of AT-II, which is associated with lung tumorigenesis. Also, recent studies have reported that *SLC34A2* plays a key role in lung cancer. *SLC34A2* expression was increased during fetal lung development and early embryonic development, but it was decreased in NSCLC tissues compared with surrounding normal lung tissue (23). In addition, studies have reported that *SLC34A2* may inhibit the growth, invasion, and migration of lung cancer cells through PI3K/Akt and Ras/Raf/MEK signaling pathways, by acting as a tumor suppressor (23, 24). In some cancers, *SLC34A2* is considered a potential prognostic marker of tumor disease. *SLC34A2* mutations in breast and thymic tumors reduce the life expectancy of patients, and *SLC34A2* overexpression was found to reduce the life expectancy of patients with brain tumors, ovarian tumors, and pancreatic tumors (25). High *SLC34A2* expression was present in approximately 75% of lung adenocarcinoma samples and was associated with significantly improved overall patient survival (26). A recent study showed that *SLC34A2* (hazard ratio: 0.86, P = 0.03, coefficient = -0.1551) could be a potential prognostic immune-related gene by validating the characteristics of immune infiltration analysis in patients with NSCLC and its correlation with survival outcomes (27). It has been revealed by other studies that the high expression of *EREG* in lung adenocarcinoma is detrimental to the survival of individuals (28). In gastric cancer, it was found that the EREG mRNA expression was an independent predictor of poor survival, and that the overall survival was significantly reduced in patients with gastric cancer with high expression (29). *DLX* homeobox genes, which belong to the NK family, play a dual role in development and cancer (30). In human ovarian cancer cells, it has been found that DLX5 is essential for regulating AKT signaling, thereby promoting cell proliferation and survival. *FEZF1*, which is a highly conserved transcription factor belonging to the C2H2 zinc finger protein family, has been shown to reduce cell proliferation and migration in human cervical cancer cell lines when it is knockdown and to act as an independent predictor of cervical cancer recurrence (31). *H2BC9*, which belongs to the H2B aggregator histone gene family, is commonly recognized as a molecular biomarker and an independent prognostic factor for *H2BC9* cervical cancer in cervical cancer and glioma (32, 33).

In conclusion, a study was conducted in which the gene mutations in lung squamous cell carcinoma with *TTN* mutations were analyzed. Ten prognostic beneficial factors of the heterogeneous expression of lung squamous cell carcinoma genes driven by *TTN* mutations were screened for and *DLX5, FEZF1, H2BC9, EREG*, and *SLC34A2* were identified as the main factors of the prognostic models. These factors were validated on external data sets. However, it was noted that a public database was used for the analysis and the screened prognostic factors were not experimentally validated. Additionally, data from a large clinical cohort was lacking for testing. It was planned that in a follow-up study, data collected from multiple cases would be analyzed, the correlation of *TTN* mutation-driven gene heterogeneous expression would be validated based on clinical trials, and the mechanism of *TTN* mutation regulation of gene expression would be investigated through cellular assays.

In conclusion, in our study, we identified *SLC34A2*, *BARX1*, and *HOXC10* as the major prognosis-related genes by constructing a prognostic model constructed by the differential expression of *TTN* mutation-driven genes through bioinformatics analysis. These findings could provide prognostic biomarkers for LUSC and possible new targets for its clinical treatment.

## Data availability statement

The datasets presented in this study can be found in online repositories. The names of the repository/repositories and accession number(s) can be found in the article/supplementary material.

## Author contributions

YZ, GZ, XP and CZ designed the study. ZL, XZ and XT completed the analysis. ZL and XT wrote the manuscript. RW proofread the manuscript and put forward constructive opinions, and all the authors have read and approved the submission of the manuscript. All authors contributed to the article and approved the submitted version.
